# Supplementary material for: Predicting B cell receptor substitution profiles using public repertoire data
Source: PLoS Comput Biol. 2018 Oct 17;14(10):e1006388. doi: 10.1371/journal.pcbi.1006388 (PMC6205660; doi:10.1371/journal.pcbi.1006388)
Supplement: S1 Text — Details of the penalized regression model. (PDF) [file pcbi.1006388.s001.pdf]

Model Interpretation

In this subsection, we provide statistical motivation for our penalized regression model, which can be interpreted as specifying an ensemble of multinomial logistic regression models at each AHo position. We use some of the notation mentioned in the methods section and introduce new notation as needed. We begin by describing the structure of the multinomial logistic regression models and then discuss how we perform model averaging with these component models to form the estimator $F(\mathbf{X})$ as stated in the methods section. We conclude this subsection by showing that our regularized minimization problem can be characterized as a maximum a posteriori (MAP) inference problem.

Suppose we observe M amino acids at the jth AHo position for the ith CF; for simplicity, we let $y_1, ..., y_M$ denote the observed amino acids. We assume that $y_1, ..., y_M$ are drawn independently from a common multinomial distribution with 20 possible categories and define a logistic regression model for the amino acid probabilities that does not include covariates. The standard way to formulate such a model is as follows:

$$\log\left(\frac{\mathrm{P}(y_m = c)}{\mathrm{P}(y_m = 20)}\right) = \beta^{(c)}, \qquad \forall c \in \{1, ..., 20\},$$

where c indexes a particular amino acid, $\beta^{(20)} \equiv 0$, and $m = 1, ..., M$. We can equivalently represent the model as:

$$\mathrm{P}(y_m = c) = \frac{\exp(\beta^{(c)})}{\sum_{c'=1}^{20} \exp(\beta^{(c')})}, \qquad \forall c \in \{1, ..., 20\},$$

where $m = 1, ..., M$. If we let $\widehat{p}_c$ denote the observed proportion of amino acid c in the sample, then it is easy to show that maximizing the multinomial likelihood of the M observations with respect to the $\beta^{(c)}$ parameters leads to the following parameter estimates:

$$\widehat{\beta}^{(c)} = \log\left(\frac{\widehat{p}_c}{\widehat{p}_{20}}\right), \qquad \forall c \in \{1, ..., 20\},$$

which implies that:

$$\widehat{\mathrm{P}}(y_m = c) = \widehat{p}_c, \qquad \forall c \in \{1, ..., 20\},$$

where $\widehat{\mathrm{P}}(y_m = c)$ represents the logistic regression estimate of $\mathrm{P}(y_m = c)$. Therefore, this multinomial logistic regression model provides simple, intuitive estimates of the amino acid probabilities. While these logistic regression estimates may seem trivial, the underlying framework allows for easy integration of CF-specific and site-specific information into our model.

The above model considers the amino acid probabilities at only one AHo position so one could fit this multinomial logistic regression model at each of the 149 AHo positions in a CF to obtain a complete substitution profile estimate. In this paper, each CF-specific substitution profile estimate is a maximum likelihood estimate (MLE) obtained by fitting 149 multinomial regression models to the observed amino acid data in the CF. Given that our proposed modeling procedure computes a per-site weighted average between the subsampled profiles $\mathbf{X}$ and all the external profile estimates $\mathbf{X}^*$, one can interpret this model $F(\mathbf{X})$ as defining an ensemble of multinomial logistic regression estimates at each AHo position.

To specify this relationship more clearly, we denote the likelihood of $\mathbf{Y}_{i,j,\bullet}$ as follows:

$$\mathbf{Y}_{i,j,\bullet} \sim \mathrm{MVN}\big(\boldsymbol{\mu}_{i,j,\bullet}, \sigma^2 \mathbb{I}_{20}\big),$$
$$\boldsymbol{\mu}_{i,j,\bullet} \equiv \sum_{l=1}^{p} \alpha_{j,l} \cdot \mathbf{X}^*_{i,j,\bullet,l} + \Big(1 - \sum_{l=1}^{p} \alpha_{j,l}\Big) \cdot \mathbf{X}_{i,j,\bullet},$$

where MVN means multivariate normal, $\boldsymbol{\mu}_{i,j,\bullet}$ signifies the mean vector of $\mathbf{Y}_{i,j,\bullet}$ and defines our ensemble model, σ^2 represents an unknown variance parameter, $\mathbb{I}_{20}$ symbolizes the 20×20 identity matrix, and p represents the number of external profiles in $\mathbf{X}^*$. Note that $\boldsymbol{\mu}_{i,j,\bullet}$ depends on the multinomial logistic regression estimates described previously as both $\mathbf{X}$ and $\mathbf{X}^*$ contain the MLE-based substitution profile estimates. In addition, the form of $\boldsymbol{\mu}_{i,j,\bullet}$ relates to our previous definition of $F(\mathbf{X})$ by observing that $\boldsymbol{\mu}_{\bullet,j,\bullet} = f(\mathbf{X}_{\bullet,j,\bullet}; \boldsymbol{\alpha}_{j,\bullet})$. We can also integrate the inequality constraints of the ensemble weights $\alpha_{j,l}$ into the likelihood function by including the indicator term $\mathbb{1}_{\boldsymbol{\alpha}} \equiv \mathbb{1}\{0 \leq \alpha_{j,l} \leq 1;\ 0 \leq \sum_{l=1}^{p} \alpha_{j,l} \leq 1;\ \forall j, l\}$. The lasso penalties can be incorporated into our model through the use of sparsity-inducing prior distributions.

Specifically, the priors placed on $\boldsymbol{\alpha}$ can be represented as:

$$\mathrm{P}(\boldsymbol{\alpha}_{\bullet,l}) \propto \exp\Big(-\lambda_1 \big|\big|\boldsymbol{\alpha}_{\bullet,l}\big|\big|_1 - \lambda_2 \big|\big|\nabla^d(\boldsymbol{\alpha}_{\bullet,l})\big|\big|_1\Big), \qquad \forall l \in \{1, ..., p\},$$

where $\lambda_1, \lambda_2 \geq 0$ and $d \in \mathbb{N}$ are the same tuning parameters specified in the methods section (Park and Casella, 2008; Kyung et al., 2010; Faulkner et al., 2018). These Laplace-like prior distributions can be expressed as scale mixtures of normal distributions with independent gamma distributed variances (Kyung et al., 2010); for a more comprehensive discussion on shrinkage priors of this form, we refer readers to (Faulkner et al., 2018).

The posterior $\mathrm{P}(\boldsymbol{\alpha}|\mathbf{Y})$ can be presented in the following manner:

$$\begin{aligned}\mathrm{P}(\boldsymbol{\alpha}|\mathbf{Y}) &\propto \mathrm{P}(\mathbf{Y}|\boldsymbol{\alpha})\mathrm{P}(\boldsymbol{\alpha}) \\ &\propto \prod_{i=1}^{500}\prod_{j=1}^{149}\mathrm{P}(\mathbf{Y}_{i,j,\bullet}|\boldsymbol{\alpha}_{j,\bullet})\prod_{l=1}^{p}\mathrm{P}(\boldsymbol{\alpha}_{\bullet,l}).\end{aligned}$$

Note that we assume the $\mathbf{Y}_{i,j,\bullet}$ vectors are independent conditional on $\boldsymbol{\alpha}_{j,\bullet}$ and the prior distribution on $\boldsymbol{\alpha}$ factorizes across $l \in \{1, ..., p\}$. The MAP estimate of the ensemble weights $\boldsymbol{\alpha}$ is obtained by maximizing $\mathrm{P}(\boldsymbol{\alpha}|\mathbf{Y})$ and is equivalent to the estimate that minimizes the regularized objective function shown in the methods section. The latter assertion can be seen as the posterior on $\boldsymbol{\alpha}$ can be monotonically transformed into our penalized minimization problem (up to a constant factor in $\boldsymbol{\alpha}$).

Of course, there are limitations to this interpretation of our modeling framework. For instance, $\mathbf{Y}_{i,j,\bullet}$ is a frequency vector, yet we model the likelihood of $\mathbf{Y}_{i,j,\bullet}$ using a multivariate normal distribution, which has support over all real numbers; we could potentially remedy this problem by modeling the likelihood of $\mathbf{Y}_{i,j,\bullet}$ as a Dirichlet distribution as its negative log-likelihood looks similar to a cross-entropy loss function. In addition, we specify priors on $\boldsymbol{\alpha}$ that also have support over the real line, which is not realistic. Our assumption that the $\mathbf{Y}_{i,j,\bullet}$ vectors are conditionally independent given $\boldsymbol{\alpha}_{j,\bullet}$ is used solely for presentation purposes and does not hold in practice because the substitution profile data in both $\mathbf{X}$ and $\mathbf{X}^*$ are correlated across AHo positions. Despite these issues with our statistical representation of the penalized regression model, our results demonstrate that the model is useful for predicting CF-specific substitution profiles in data-sparse situations.
