# Supplementary material for: Predicting B cell receptor substitution profiles using public repertoire data
Source: PLoS Comput Biol. 2018 Oct 17;14(10):e1006388. doi: 10.1371/journal.pcbi.1006388 (PMC6205660; doi:10.1371/journal.pcbi.1006388)
Supplement: S2 Text — Explanation of the continuous approximation of the Jaccard similarity used in the optimization. (PDF) [file pcbi.1006388.s002.pdf]

Smoothed Jaccard Similarity

As we stated in the methods section, optimization on the Jaccard similarity objective function is difficult because this metric is locally flat with respect to our parameter values of $\boldsymbol{\alpha}$. For some small changes in $\boldsymbol{\alpha}$, the averaged Jaccard similarity can remain at the same value because the Jaccard sets continue to hold the same elements. This is a problem because the L-BFGS-B optimization algorithm uses gradient information to determine its search direction for $\boldsymbol{\alpha}$ and the Jaccard similarity gradients are often zero due to the reasoning given above, which results in premature termination of the L-BFGS-B optimizer. We now describe an approach to "smooth" the Jaccard similarity objective function that directly addresses these concerns.

For notational simplicity, we let $\{a_i\}_{i=1:20}$ and $\{b_i\}_{i=1:20}$ denote the actual and predicted amino acid frequencies, respectively, at a particular AHo position for a given CF. As before, t represents the cutoff separating high versus low frequency amino acids. We also introduce the following indicator function $f(a,t) \equiv \mathbb{1}\{a \geq t\}$ for any amino acid frequency a. If we further let $A = \mathcal{A}(\{a_i \mid a_i \geq t\})$ and $B = \mathcal{A}(\{b_i \mid b_i \geq t\})$ with $\mathcal{A}(\cdot)$ as defined in the methods section, then the Jaccard similarity between sets A and B can be rewritten as:

$$J(A,B) \equiv \frac{|A \cap B|}{|A \cup B|} = \frac{\sum_{i=1}^{20} f(a_i,t)f(b_i,t)}{\sum_{i=1}^{20} \min\{1, f(a_i,t) + f(b_i,t)\}}.$$

The local flatness of the Jaccard similarity objective is due to the constant regions of $f(a_i,t)$ and the non-smooth curvature of $f(a_i,t)$ at the jump point t. It turns out that $f(a_i,t)$ can also be described as the limit of the following function:

$$f_\epsilon(a_i,t) = \frac{1}{1+e^{-\epsilon(a_i-t)}},$$

as $\epsilon \to \infty$. Thus, to obtain a "smooth" transformation of $J(A,B)$, we replace $f(a_i,t)$ with $f_\epsilon(a_i,t)$ in the above equation of $J(A,B)$ and set ϵ (i.e. the steepness parameter) to be a small number. Figure **??** plots the function $f_\epsilon(a_i,0.2)$ against $a_i \in [0,1]$ for various values of ϵ.

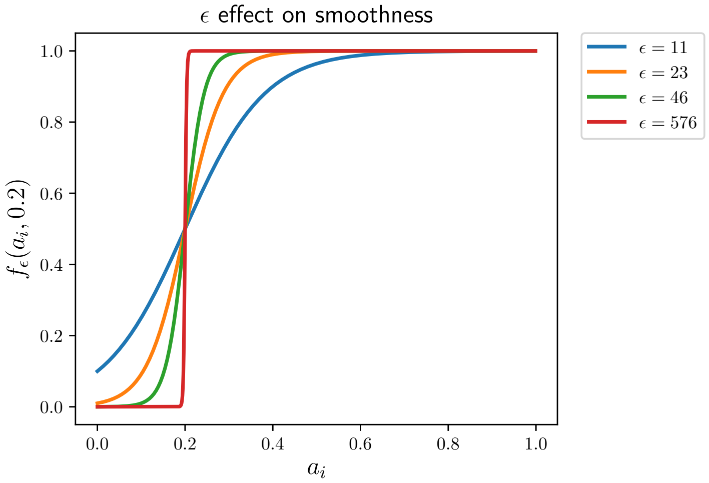

S12 Fig. A plot of the function $f_\epsilon(a_i,0.2)$ against $a_i \in [0,1]$ for various values of ϵ. As ϵ gets larger, $f_\epsilon(a_i,0.2)$ tends to the indicator function $f(a_i,0.2)$.

Fortunately, the use of this "smooth" Jaccard similarity function allows the L-BFGS-B optimization algorithm to converge properly. To use this "smoothed" objective function in the right manner, we were interested in finding the largest values of ϵ that still permitted proper L-BFGS-B convergence. We utilized $\epsilon = 23$ throughout all our Jaccard similarity experiments because we found that this value of ϵ satisfied our selection criteria specified previously.
