# Supplementary material for: Predicting B cell receptor substitution profiles using public repertoire data
Source: PLoS Comput Biol. 2018 Oct 17;14(10):e1006388. doi: 10.1371/journal.pcbi.1006388 (PMC6205660; doi:10.1371/journal.pcbi.1006388)
Supplement: S3 Text — References used in the Supporting Information sections. (PDF) [file pcbi.1006388.s003.pdf]

References

1. Park T, Casella G. The Bayesian lasso. Journal of the American Statistical Association. 2008;103(482):681–686.
2. Kyung M, Gill J, Ghosh M, Casella G, et al. Penalized regression, standard errors, and Bayesian lassos. Bayesian Analysis. 2010;5(2):369–411.
3. Faulkner JR, Minin VN, et al. Locally adaptive smoothing with Markov random fields and shrinkage priors. Bayesian Analysis. 2018;13(1):225–252.
