# Supplementary figures and images for: Predicting B cell receptor substitution profiles using public repertoire data
Source: PLoS Comput Biol. 2018 Oct 17;14(10):e1006388. doi: 10.1371/journal.pcbi.1006388 (PMC6205660; doi:10.1371/journal.pcbi.1006388)

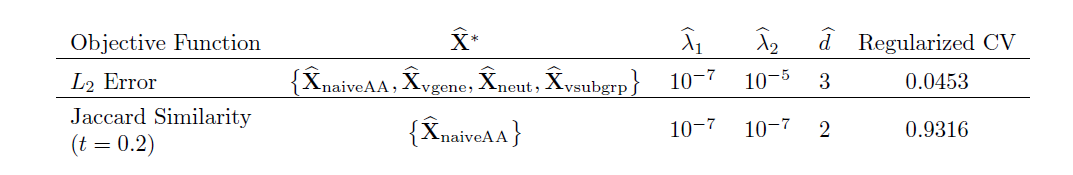

Supplement: S1 Table — We present the optimal tuning parameters selected from λ1, λ2 = 10−7, 5.05 × 10^−6^, 10−5 and d = 1, 2, 3 and show the associated cross-validated performance estimates. Note that the possible choices of X* for the L2 error metric include the {X^naiveAA,X^vgene,X^neut} and {X^naiveAA,X^vgene,X^neut,X^vsubgrp} groupings, while the {X^naiveAA} and {X^naiveAA,X^vgene} groupings are the possible X* choices for the smoothed Jaccard similarity objective. (TIFF) [file pcbi.1006388.s004.tiff]

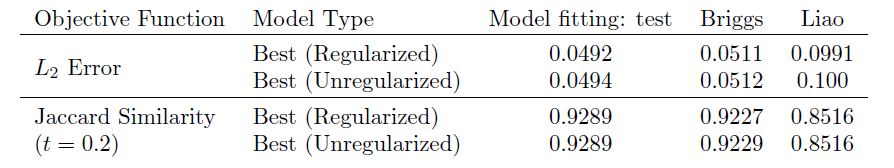

Supplement: S2 Table — The unregularized and regularized model performance using either L2 Error or Jaccard Similarity resulting from predicting on independent datasets. We provide results for the testing portion of the model fitting dataset, the Briggs validation dataset, and the Liao dataset. Note that the term “baseline” refers to predictions made using only the input sequence (i.e. model predictions with all parameter values of α set to 0) and lower L2 error and higher Jaccard Similarity is preferred. (TIFF) [file pcbi.1006388.s005.tiff]

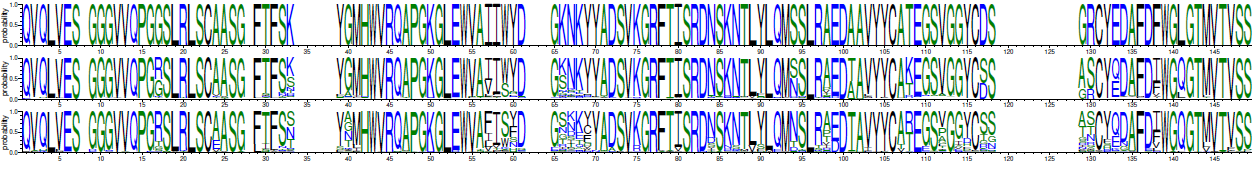

Supplement: S2 Fig — The logos are plotted using AHo numbers (1-149) and AHo positions undefined in the sequence are shown as empty columns. The predicted profile (middle) captures much of the amino acid composition information associated with the full profile (bottom). (TIFF) [file pcbi.1006388.s007.tiff]

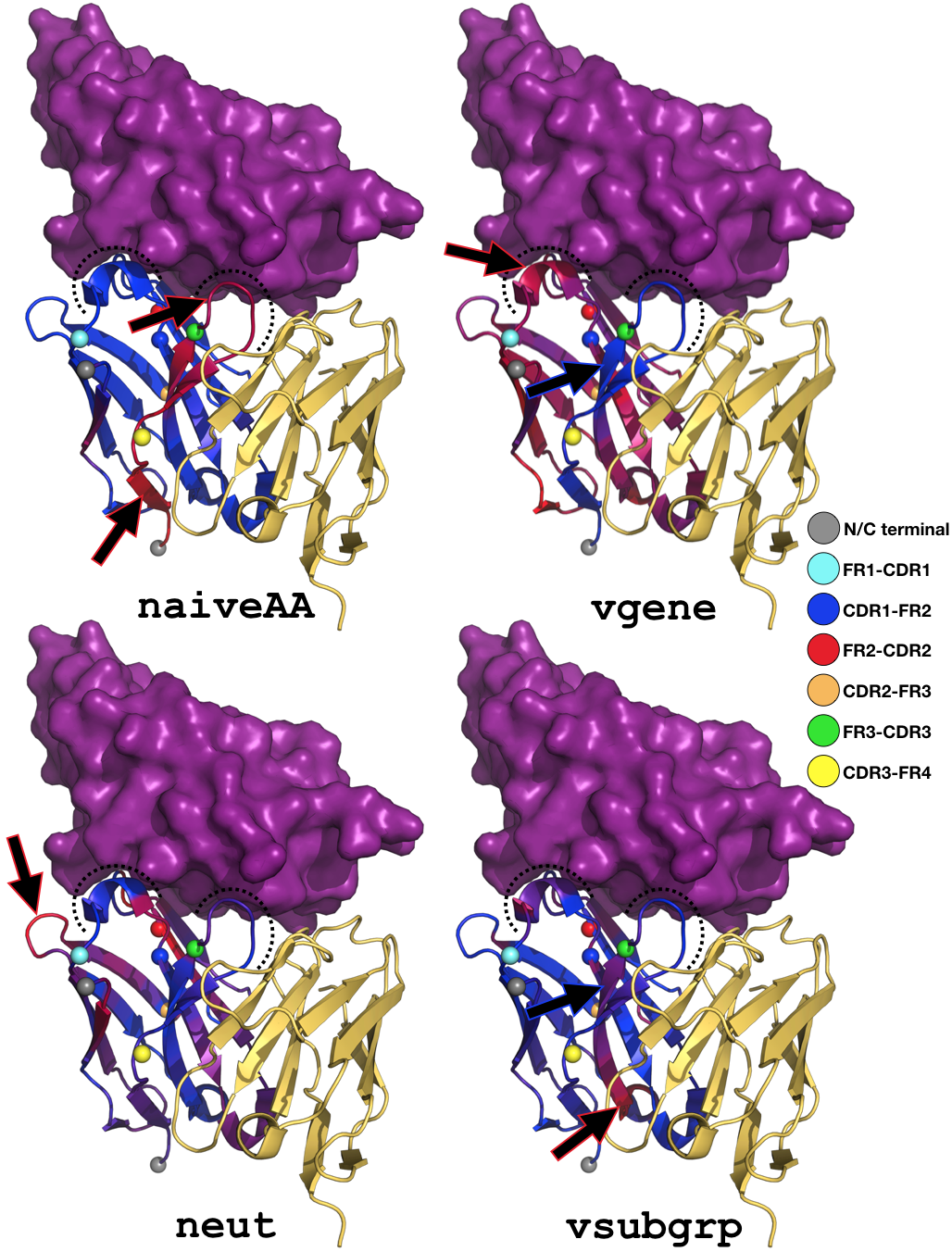

Supplement: S3 Fig — The antigen (PD-L1) appears as a purple surface at the top of the images, the light chain appears in yellow cartoon, and the heavy chain is displayed using a blue to red color gradient. The color gradient represents the possible values of profile weights in α and goes from blue at a zero weight to red at the maximum weight for the profile. The black dashed lines mark the CDR loops; note that the CDR2 loop is hidden behind the CDR1. The colored balls represent the AHo-defined FWK/CDR boundaries. The black arrows indicate regions of high profile weight. The X^naiveAA profile is heavily weighted in CDR3 and FWK4. The X^vgene profile weighting is fairly even from FWK1 through FWK3; it spikes slightly in CDR1 and completely disappears beyond FWK3, which is expected as the V-D junction region starts past the end of FWK3. The X^neut profile weighting is fairly even across sites but spikes near the beginning of FWK3 (the “outer” loop). The X^vsubgrp profile weighting is distributed similarly to that of the X^vgene profile with the exception of a spike at the end of FWK3 (i.e. at the heavy and light chain interface). (TIFF) [file pcbi.1006388.s008.tiff]

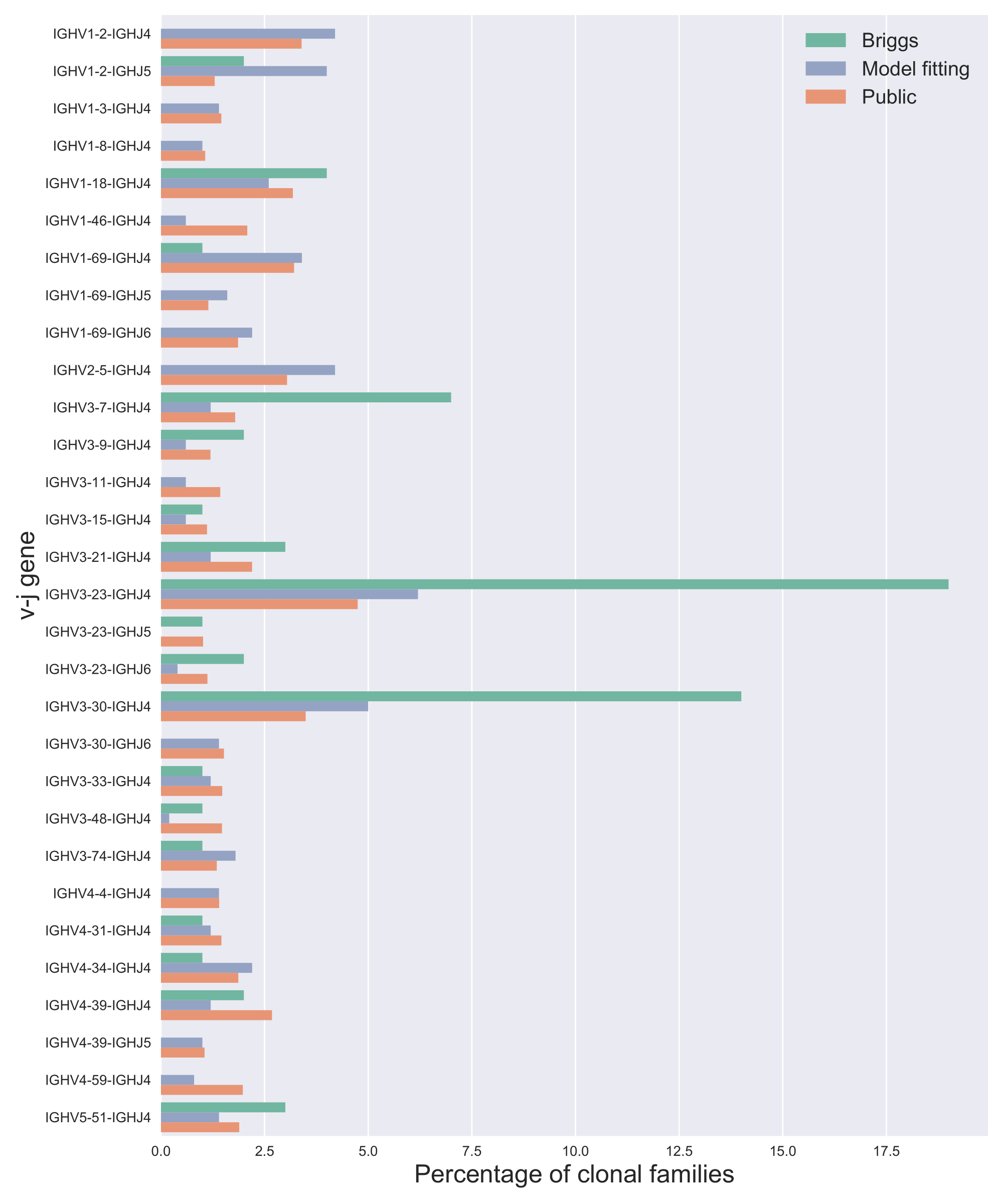

Supplement: S4 Fig — Minimum frequency of 1% in either partition used as a cutoff for inclusion. (TIFF) [file pcbi.1006388.s009.tiff]

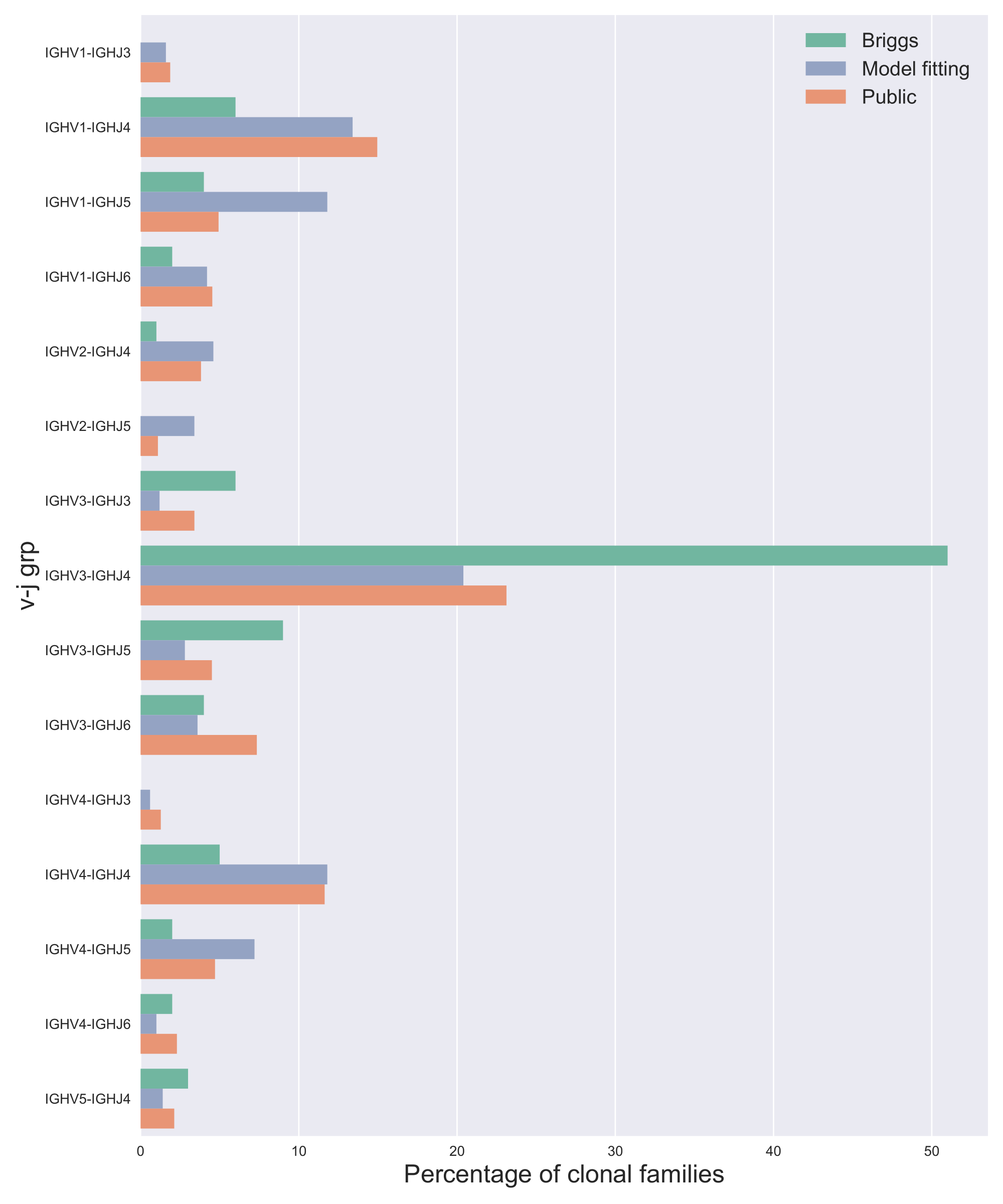

Supplement: S5 Fig — Minimum frequency of 1% in either partition used as a cutoff for inclusion. (TIFF) [file pcbi.1006388.s010.tiff]

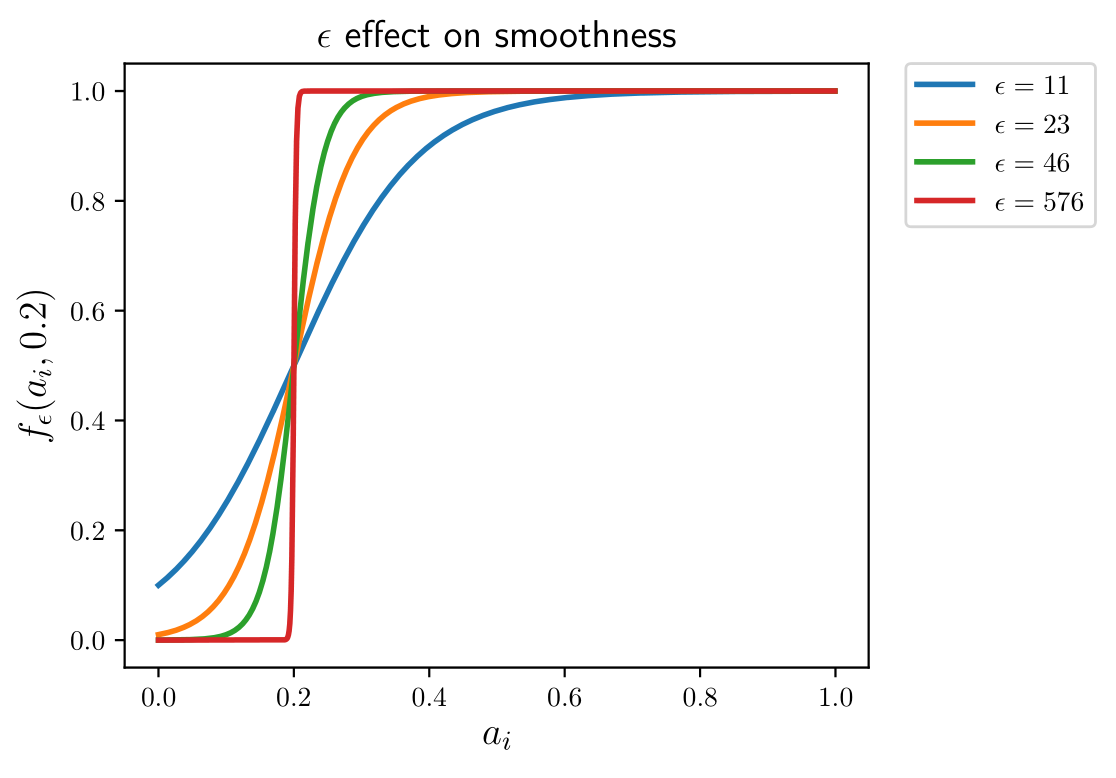

Supplement: S8 Fig — As ϵ gets larger, fϵ(ai, 0.2) tends to the indicator function f(ai, 0.2). (TIFF) [file pcbi.1006388.s013.tiff]
